# Left atrial appendage thrombus in an elderly patient with atrial fibrillation during non-cardiac surgery- a case report-

**DOI:** 10.1186/s12871-023-02286-8

**Published:** 2023-10-06

**Authors:** Yaru Li, Luyang Jiang, Lu Wang, Xinrui Yin, Qiaoyu Han, Yi Feng

**Affiliations:** Department of Anesthesiology, Peking University People’s Hospital, 11 Xi Zhi Men South Street, Beijing, 100044 China

**Keywords:** Left atrial appendage, Thrombosis, Transesophageal echocardiography, Monitoring, Intraoperative

## Abstract

**Background:**

Perioperative newly developed left atrial appendage (LAA) thrombus is a rare but extremely challenging event for anesthesia management. It’s important to identify and diagnose thrombosis in high-risk populations promptly.

**Case presentation:**

In the case of an elderly patient with atrial fibrillation undergoing non-cardiac surgery, we recorded the findings before and after thrombosis during the operation using transesophageal echocardiography (TEE). Through timely detection of the thrombus and proactive management, a satisfactory therapeutic effect was ultimately achieved.

**Conclusions:**

Clinicians should be aware of the potential risk of LAA clot formation during surgery, even if it was not detected preoperatively. And TEE is valuable for monitoring newly developed perioperative thrombosis.

**Supplementary Information:**

The online version contains supplementary material available at 10.1186/s12871-023-02286-8.

## Background

The left atrial appendage (LAA) is a finger-like projection originating from the left atrium (LA). It is primarily formed by adhesion of the primitive left upper pulmonary vein. The multiple-lobular structure and intrinsic pectinate muscles make it prone to clot formation. More than 90% of left atrial thrombus arise from the LAA [1]. In a meta-analysis comprising thirty-five studies and a total of 14,653 patients, the incidence of non-paroxysmal atrial fibrillation-related thromboembolism was found to be 4.81% [2]. Newly formed LAA thrombus during surgery can be exceedingly dangerous and may result in postoperative stroke. Therefore, timely detection and diagnosis are imperative. The present report details a case involving an elderly patient who was receiving anticoagulant therapy and was found to have a newly thrombus formation during surgery, as detected by TEE examination. Following active treatment, the patient recuperated and was subsequently discharged. This case underscores the significance of new thrombotic events during surgery and serves to highlight the vigilance required by anesthesiologists. The report has obtained informed consent from the patient.

## Case presentation

An 84-year-old female patient with a body mass index (BMI) of 26.2 kg/cm^2^ was admitted to the hospital due to the mucus and bloody stool. The patient had a 20-year history of hypertension and a 10-year history of atrial fibrillation. She was taking betaloc 25 mg and aspirin 100 mg daily. Two years ago, she suffered a stroke which left her with speech impairment. Upon admission, her blood pressure was 135/70 mmHg and her heart rate was 105 bpm. The preoperative diagnosis was pelvic mass with invasion of the uterus and rectum. The preoperative transthoracic electrocardiogram (TTE) showed that the left atrial transverse diameter was 4.7 cm, the left ventricular mass (indexed to body surface area) was 130 g, the left ventricular end-systolic diameter was 2.8 cm, and the left ventricular end-diastolic diameter was 4.3 cm. Meanwhile, the TTE report indicated that the patient had hypertrophic cardiomyopathy, mild regurgitation of the mitral and tricuspid valves, and a slight increase in pulmonary artery pressure (estimated at 42mmHg). The preoperative D-Dimer level was 572 ng/ml. The planned procedure involved hysterectomy with double adnexectomy, rectal resection, and enterostomy.

**Fig. 1:**
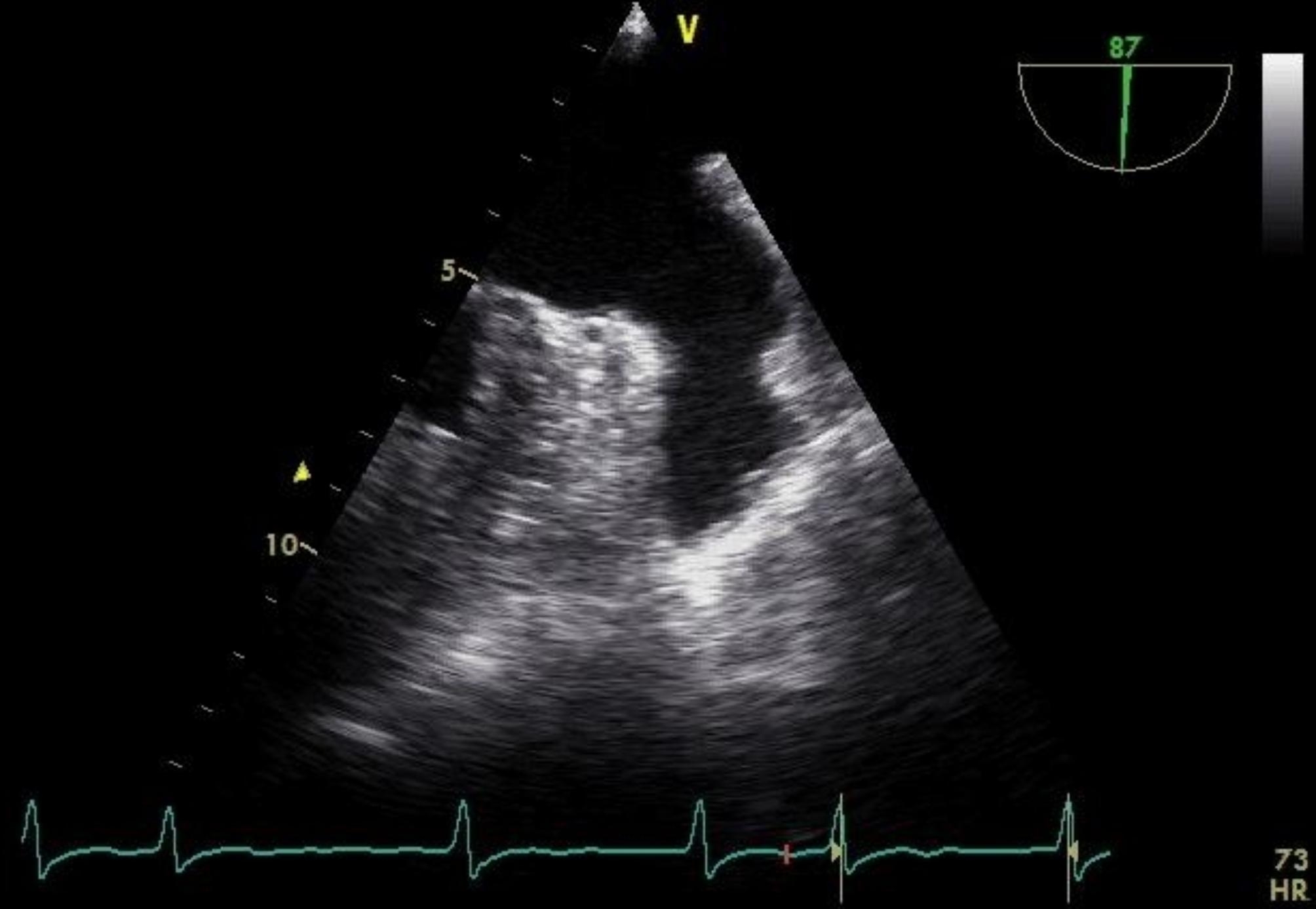
Clear left atrial appendage preoperatively

**Fig. 2:**
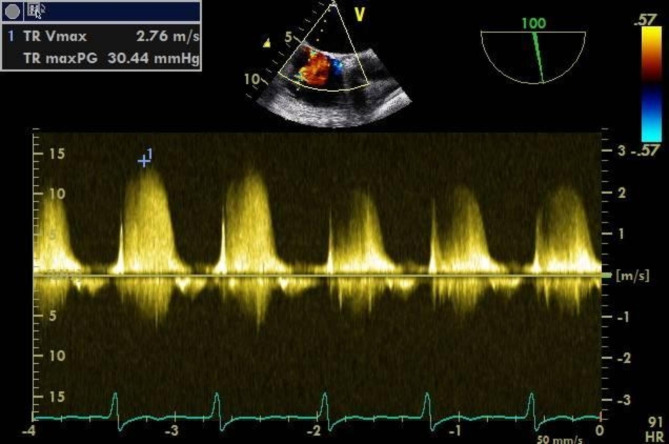
Tricuspid regurgitation

**Fig. 3:**
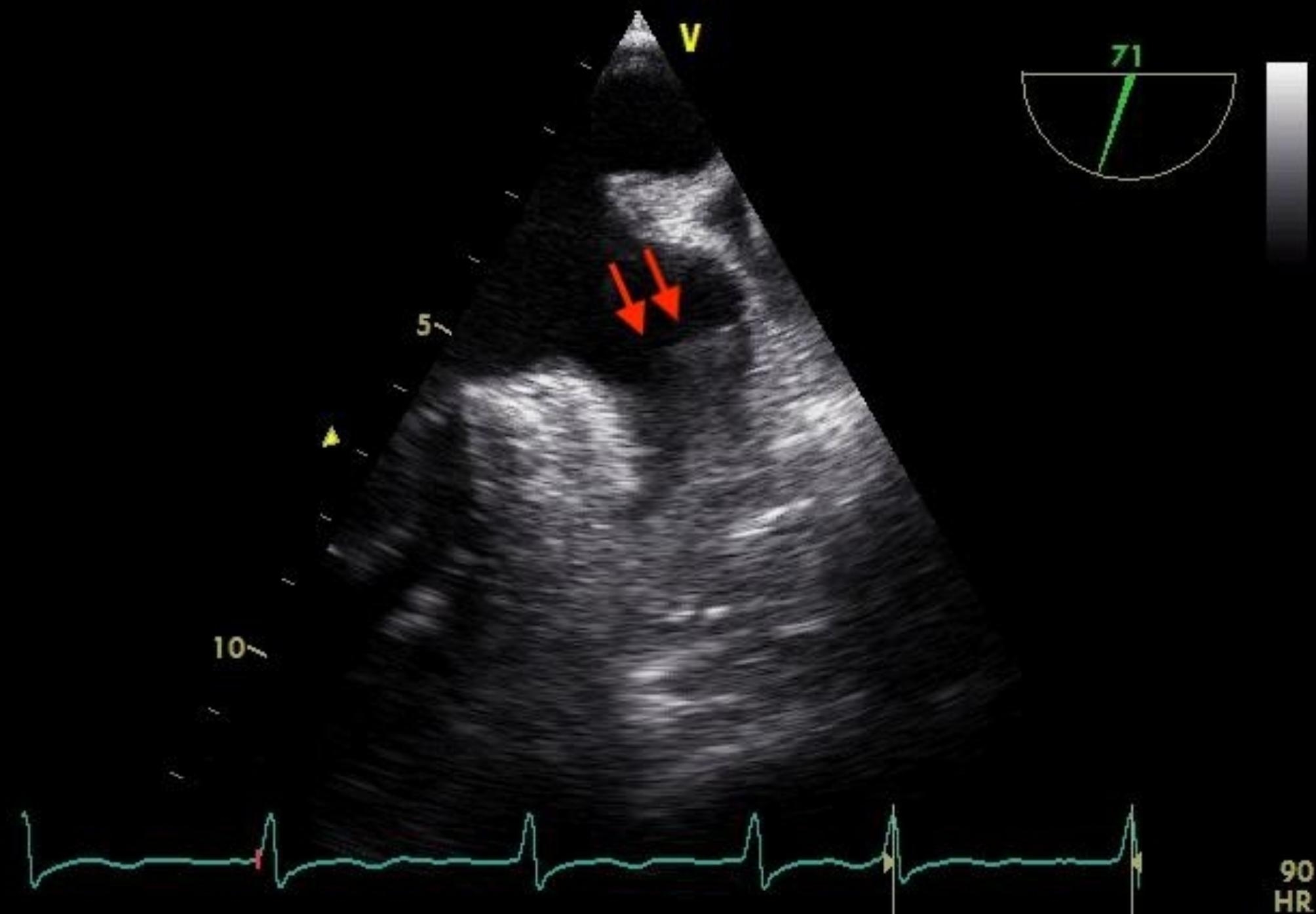
New left atrial appendage thrombus intraoperatively

**Fig. 4:**
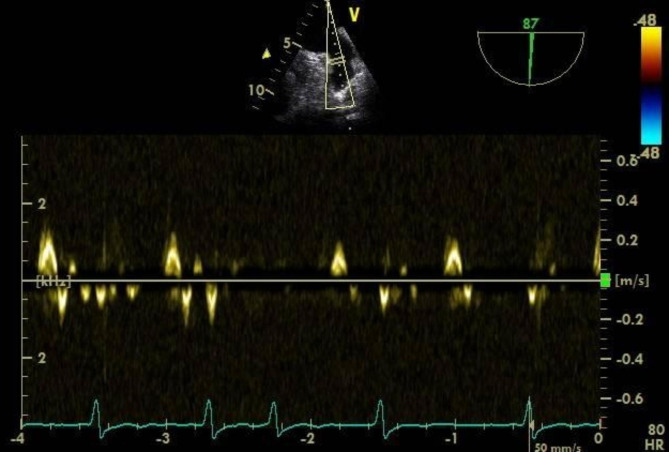
Measurement of left atrial appendage emptying velocity by pulse Doppler. The emptying velocity of left atrial appendage was 20 cm/s

**Fig. 5:**
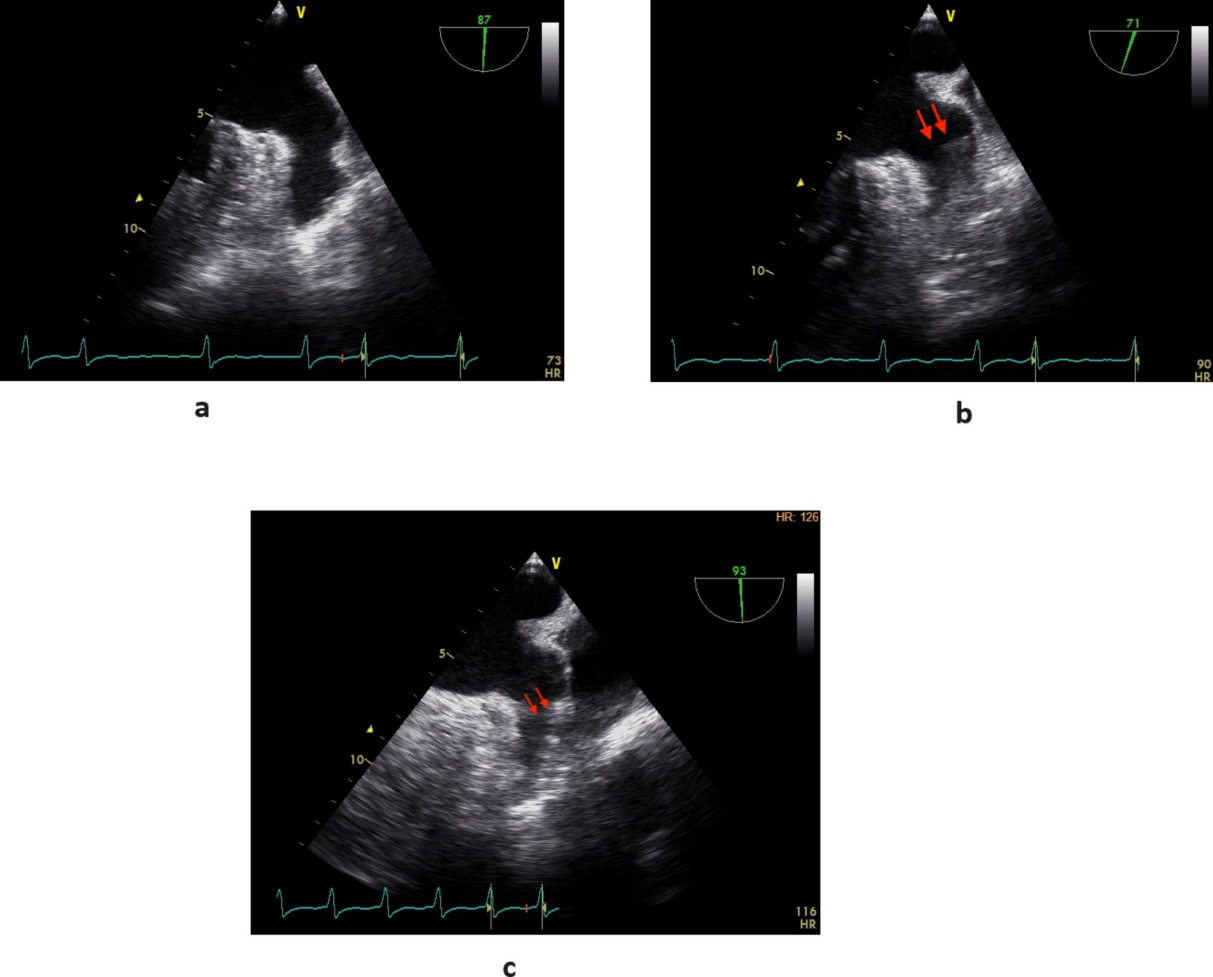
Comparison of the left atrial appendage preoperatively, intraoperatively, pre-anticoagulation and post-anticoagulation. **A** Preoperative: clear and unobstructed left atrial appendage. **B** Pre-anticoagulation intraoperatively: new thrombus in the left atrial appendage (red arrow). **C** Post-anticoagulation intraoperatively: reduced and blurred thrombus in the left atrial appendage (red arrow)

After a comprehensive assessment and preparation including low-molecular-weight heparin bridging therapy (enoxaparin 1 mg/kg twice daily for 5 days), the patient had been scheduled for surgery. The electrocardiogram upon admission to the operating room exhibited atrial fibrillation with heart rate of 98 bpm and blood pressure of 132/65 mmHg. Following appropriate local anesthesia, arterial and central venous catheterization were performed. Thereafter, anesthesia was induced with 1.5 mg of midazolam, 14 mg of etomidate, 30 ug of sufentanil, and 8 mg of cisatracurium besylate. Taking into account the patient’s hypertrophic cardiomyopathy, following induction, an esophageal ultrasound probe (6TC-RS GE Medical Horton, Norway) was gently inserted orally to meticulously monitor ventricular function. To routine inspect the left atrial appendage, no significant thrombus or spontaneous echo contrast (SEC) were observed (Fig. 1 and Video 1). However, subsequent examinations revealed myocardial hypertrophy, the hypertrophy segment of which was in apical segment. (Video 2), mild mitral and aortic regurgitation (Video 3), and moderate-to-severe tricuspid regurgitation (TR Vmax was 276 cm/s, Fig. 2 and Video 4). The patient’s blood pressure was mildly reduced and maintained between 110–140/50–70 mmHg with minimal vasopressor support, while the heart rate was kept below 90 bpm. However, 3 h into the surgery, the patient’s heart rate significantly increased (over 100 bpm), and the blood pressure dropped, necessitating higher doses of vasoactive medication. At this time, TEE examination revealed a significant jelly-like fresh thrombus in the LAA (Fig. 3 and Video 5) with a peak LAA emptying velocity of approximately 20 cm/s by Pulse Wave Doppler (Fig. 4). As the surgery was concluding, we only slightly increased the heart rate to reduce blood stasis in the LAA and did not perform any other treatment. Approximately half an hour later, after hemostasis was achieved, anticoagulation therapy was recommended by anesthesiologist, and 2500 IU of heparin (about 40IU/Kg) was administered intravenously. After 20 min, the size of the thrombus decreased and the shape became blurred (Video 6). The left atrial appendage views before and after anticoagulation therapy are depicted in Fig. 5. Subsequently, the patient was transferred to the intensive care unit (ICU), where extubated two days later without any complication. Following the administration of standard anticoagulation therapy, involving a transition from low molecular weight heparin to oral medication, the patient was discharged from the hospital 10 days post-surgery. And the patient has been advised to take 3 mg of warfarin orally per day after the surgery for the long term.

## Discussion and conclusions

Patients with atrial fibrillation are still at risk of developing thrombosis even when receiving anticoagulant therapy. Therefore, there are some tools in clinical practice to evaluate the risk of thrombosis. Among them, the CHA_2_DS_2_-VASc scoring system can quickly assess the risk of thrombosis in patients. The CHA_2_DS_2_-VASc score constitutes a tool implemented in the assessment of stroke risk in non-valvular atrial fibrillation patients [3]. Our patient presented a CHA_2_DS_2_-VASc score of 6, indicating a significant risk of stroke. Although the patient received anticoagulation therapy before surgery and the operation lasted only 3 h, an unexpected thrombosis developed intraoperatively. While preoperative scale assessment holds significant importance, prompt identification and diagnosis of thrombus is equally crucial. TEE is the gold standard for diagnosing LAA thrombus with the reported sensitivity ranges between 93 and 100%, and specificity ranges between 99 and 100% [4]. The left atrial appendage view of TEE enables visualization of the LAA [5]. Spontaneous echo contrast (SEC) observed in TEE serves as a precursor to thrombus formation. Moreover, LAA emptying velocity < 40 cm/s is associated with an increased risk of SEC. When this velocity falls below 20 cm/s, the risk of thromboembolism is even greater [6]. In the current case, considering the patient’s advanced age and multiple comorbidities, we placed a TEE preoperatively for comprehensive perioperative monitoring. Subsequently, when a thrombus event occurred, the TEE proved invaluable in detecting a conspicuous thrombus shadow in the left atrial appendage. Notably, the patient’s LAA emptying velocity was measured to be approximately 20 cm/s. These findings have significant implications for the management of the patient’s postoperative care. However, the LAA body is often anatomically variable, curved or spiral-shaped, there may be some confusing differences in observing the LAA through TEE. Thus, Yu S et al. assert that cardiac computed tomography scanning has greater value in diagnosing LAA thrombus and can substitute for TEE [7]. Nevertheless, TEE holds an irreplaceable advantage during new emergency events in the operating room. In this case, TEE showed that the thrombus appeared like a fresh jelly that wouldn’t pose a big risk of embolization if it fell off. But if the clot hardened and untreated, there would cause postoperative embolization, which is very dangerous for elderly patients with a history of stroke [8]. According to the guidelines, we applied low-dose unfractionated heparin for anticoagulation therapy instead of thrombolysis as soon as hemostasis is achieved [9, 10]. In populations of such high-risk individuals, the preoperative placement of TEE allows us to observe the dynamic changes, enabling prompt identification and management of the cause of intraoperative hemodynamic instability, re-evaluation of prior TEE finding for interval change [11]. Overall, TEE provided important information and facilitated timely judgments and surgical decisions. Reports indicate that major complications related to TEE in ambulatory, non-operative settings range from 0.2 to 0.5%. The estimated mortality associated with TEE is less than 0.01% [12].

In conclusion, TEE is a valuable and safe tool for the rapid identification and diagnosis of new thrombosis during surgery. Consequently, anesthesiologists with TEE operational and interpreting qualifications are imperative for monitoring critically ill patients in non-cardiac surgeries.

### Electronic supplementary material

Below is the link to the electronic supplementary material.

Supplementary Material 1

Supplementary Material 2

Supplementary Material 3

Supplementary Material 4

Supplementary Material 5

Supplementary Material 6

## Data Availability

Not applicable.

## Abbreviations

LAA: Left atrial appendage
TEE: Transesophageal echocardiography
TTE: Transthoracic echocardiography
BMI: Body mass index
SEC: Spontaneous echo contrast

## References

[1] Beigel R, Wunderlich NC, Ho SY, Arsanjani R, Siegel RJ (2014). The left atrial appendage: anatomy, function, and noninvasive evaluation. JACC Cardiovasc Imaging.

[2] Lurie A, Wang J, Hinnegan KJ, McIntyre WF, Belley-Cote EP, Amit G (2021). Prevalence of left atrial Thrombus in anticoagulated patients with Atrial Fibrillation. J Am Coll Cardiol.

[3] Lip GY, Nieuwlaat R, Pisters R, Lane DA, Crijns HJ (2010). Refining clinical risk stratification for predicting stroke and thromboembolism in atrial fibrillation using a novel risk factor-based approach: the euro heart survey on atrial fibrillation. Chest.

[4] Zhan Y, Joza J, Al Rawahi M, Barbosa RS, Samuel M, Bernier M (2018). Assessment and Management of the left atrial appendage Thrombus in patients with Nonvalvular Atrial Fibrillation. Can J Cardiol.

[5] Hahn RT, Abraham T, Adams MS, Bruce CJ, Glas KE, Lang RM (2013). Guidelines for performing a comprehensive transesophageal echocardiographic examination: recommendations from the American Society of Echocardiography and the Society of Cardiovascular Anesthesiologists. J Am Soc Echocardiogr.

[6] Melillo E, Palmiero G, Ferro A, Mocavero PE, Monda V, Ascione L. Diagnosis and management of Left Atrium appendage thrombosis in Atrial Fibrillation Patients undergoing cardioversion. Med (Kaunas). 2019; 55. 10.3390/medicina55090511 PMC6780583 31438560

[7] Yu S, Zhang H, Li H (2021). Cardiac Computed Tomography Versus Transesophageal Echocardiography for the detection of left atrial appendage Thrombus: a systemic review and Meta-analysis. J Am Heart Assoc.

[8] Chen Y, Wright N, Guo Y, Turnbull I, Kartsonaki C, Yang L (2020). Mortality and recurrent vascular events after first incident stroke: a 9-year community-based study of 0.5 million chinese adults. Lancet Glob Health.

[9] Douketis JD, Spyropoulos AC, Murad MH, Arcelus JI, Dager WE, Dunn AS (2022). Perioperative Management of Antithrombotic Therapy: an american college of chest Physicians Clinical Practice Guideline. Chest.

[10] Ortel TL, Neumann I, Ageno W, Beyth R, Clark NP, Cuker A (2020). American Society of Hematology 2020 guidelines for management of venous thromboembolism: treatment of deep vein thrombosis and pulmonary embolism. Blood Adv.

[11] Nicoara A, Skubas N, Ad N, Finley A, Hahn RT, Mahmood F (2020). Guidelines for the Use of Transesophageal Echocardiography to Assist with Surgical decision-making in the operating room: a surgery-based Approach: from the American Society of Echocardiography in collaboration with the Society of Cardiovascular Anesthesiologists and the Society of thoracic surgeons. J Am Soc Echocardiogr.

